# Periprosthetic Joint Infections Caused by *Candida* Species—A Single-Center Experience and Systematic Review of the Literature

**DOI:** 10.3390/jof8080797

**Published:** 2022-07-29

**Authors:** Dariusz Grzelecki, Aleksandra Grajek, Piotr Dudek, Łukasz Olewnik, Nicol Zielinska, Petr Fulin, Maria Czubak-Wrzosek, Marcin Tyrakowski, Dariusz Marczak, Jacek Kowalczewski

**Affiliations:** 1 Department of Orthopedics and Rheumoorthopedics, Centre of Postgraduate Medical Education, Professor Adam Gruca Orthopedic and Trauma Teaching Hospital, Konarskiego 13, 05-400 Otwock, Poland; piotrek.dudek@gmail.com (P.D.); darmarczak@poczta.onet.pl (D.M.); jackow@o2.pl (J.K.); 2 Central Laboratory of Professor Adam Gruca Orthopedic and Trauma Teaching Hospital, Konarskiego 13, 05-400 Otwock, Poland; olagrajek@tlen.pl; 3 Department of Anatomical Dissection and Donation, Medical University of Lodz, Żeligowskiego 7/9, 90-752 Lodz, Poland; lukasz.olewnik@umed.lodz.pl (Ł.O.); nicol.zielinska@stud.umed.lodz.pl (N.Z.); 4 1^st^ Department of Orthopaedics, First Faculty of Medicine of Charles University and Motol University Hospital, V Uvalu 84, 15006 Prague, Czech Republic; petrfulin@gmail.com; 5 Department of Spine Diseases and Orthopedics, Centre of Postgraduate Medical Education, Professor Adam Gruca Orthopedic and Trauma Teaching Hospital, Konarskiego 13, 05-400 Otwock, Poland; czubakwrzosek@gmail.com (M.C.-W.); marcintyrak@gmail.com (M.T.)

**Keywords:** periprosthetic joint infections, fungal pathogens, *Candida albicans*, *Candida parapsilosis*

## Abstract

Background: The aim of this study was to analyze the treatment results of fungal periprosthetic joint infections (PJI) caused by *Candida* species from a single orthopedic center and to compare them with reports from other institutions. Methods: Eight patients operated on from January 2014 to December 2021 met the inclusion criteria and were analyzed in terms of clinical outcomes. A systematic review of the literature identified 153 patients with *Candida* PJIs extracted from 12 studies according to the PRISMA (Preferred Reporting Item for Systematic Reviews and Meta-Analyses) guidelines. Results: The success rate of the treatment in the case series was 50%. The most frequent pathogens were *Candida albicans* (three cases; 37.5%) and *Candida parapsilosis* (three cases; 37.5%). In one patient (12.5%), bacterial co-infection was noted, and in five patients (62.5%) significant risk factors of PJI were confirmed. The overall success rate on the basis of data collected for systematic review was 65.5%. A sub-analysis of 127 patients revealed statistically significant differences (*p* = 0.02) with a higher success rate for the knees (77.6%) than for the hips (58%). In 10 studies the analysis of risk factors was performed and among 106 patients in 77 (72.6%) comorbidities predispose to fungal PJI were confirmed. Bacterial co-infection was noted in 84 patients (54.9%). In 93 patients (60.7%) *Candida albicans* was the culprit pathogen, and in 39 patients (25.5%) *Candida parapsilosis* was the culprit pathogen. Based on these two most frequent *Candida* species causing PJI, the success rate of the treatment was statistically different (*p* = 0.03), and was 60.3% and 83.3%, respectively. The two-stage strategy was more favorable for patients with *Candida parapsilosis* infections (94.4% success rate) than the one-stage protocol (50% success rate; *p* = 0.02); as well as in comparison to the two-stage treatment of *Candida albicans* (65% success rate; *p* = 0.04). Conclusions: The analysis of the literature showed no differences in the overall success rate between one- and two-stage surgical strategies for all *Candida* species, but differed significantly comparing the two most frequent strains and concerning PJI localization. The frequent presence of bacterial co-infections makes it necessary to consider the additional administration of antibiotics in the case of fungal PJI.

## 1. Introduction

Chronic periprosthetic joint infections (PJI) caused by fungal pathogens are rare complications of total joint arthroplasty (TJA). Currently, the incidence constitutes around 1% of all PJIs, which corresponds to 0.005–0.02% of all infections following the primary total hip (THA) and knee arthroplasties (TKA) [1]. This problematic complication mostly concerns patients with immunosuppression including acquired immunodeficiency syndrome (AIDS), those undergoing chronic glucocorticoid therapy, with malignancies, those with deep neutropenia, and extensive skin injury (e.g., burn wounds, bedsores, and chronic wounds) [2,3].

With an increasing number of primary and revision TJAs worldwide, the absolute number of infections caused by fungal pathogens is growing proportionally. The diagnosis of fungal PJI still remains a clinical challenge due to low-grade onset, slow progress and the appearance of clinical symptoms (e.g., swelling, pain) with or without the increase of inflammatory markers [4]. 

Another aspect is the more difficult microbiological identification and culturing of the material collected during the revision surgery (synovial fluid, tissues, fluid after implant sonication). Different species of fungi need specific substrates and a longer time of culturing, even up to four weeks [5]. This implicates problems with the rapid initiation of the proper treatment. Currently, no specific guidelines for the management of fungal PJI have been established. Thus, the diagnostic and surgical protocols designed for bacterial PJIs are usually applied [6]. 

*Candida* species are the most common fungal pathogens identified in approximately 85% of all mycological cultures [7]. Among these, *Candida albicans* and *Candida parapsilosis* are the most frequent isolates (55–65% and 13–33%, respectively) [8]. 

Most studies describing the clinical outcomes and different treatment strategies of fungal PJI are based on small groups of patients or are case reports [9,10,11,12]. The analyses performed on larger cohorts concern data collected from several centers [13,14,15], or from systematic reviews [16,17]. Currently, two-stage revisions are known as the gold standard for the treatment of bacterial PJI and were also reported as a preferable protocol in those caused by fungal pathogens [7,10,18,19,20]. However, one-stage surgical strategies are also performed with good results in different orthopedic centers [21,22]. Recently, several valuable studies concerning *Candida* PJIs were published [12,23,24,25,26] but not included in currently available systematic reviews [17,27]. 

That is why an up-to-date systematic review presenting various protocols and their outcomes in the treatment of fungal PJIs caused by *Candida* seems to be of great importance for both the literature and especially for everyday clinical practice.

Thus, the aims of this study were: (1) to analyze patients with fungal PJI caused by *Candida* species following THA or TKA in a single orthopedic center, and (2) to systematically review the literature concerning PJIs due to *Candida* species following THA or TKA in order to suggest the optimal treatment protocols of these complications. 

## 2. Materials and Methods

### 2.1. Case Series

This retrospective analysis included all patients with chronic fungal PJI caused by *Candida* species, diagnosed and treated in a single orthopedic center from January 2014 to December 2021. PJIs were recognized according to the International Consensus Meeting (ICM) 2013 and ICM 2018 definitions depending on the time of patient hospitalization [28,29]. Only patients with confirmed fungal PJI, according to the ICMs criteria and with the mycological identification, were included in the analysis. Demographic (age, sex, BMI) and clinical data such as: operated joint, time from primary TJA to PJI, risk factors of PJI (e.g., diabetes, alcoholism, drug abuse, use of immunosuppressive agents, chronic inflammatory diseases, sepsis, severe prior infections and multiple surgical procedures on the operated joint), laboratory parameters (serum C-reactive protein concentration [CRP] and erythrocyte sedimentation rate [ESR]), treatment protocols, antifungal agents and final outcomes were extracted from the hospital electronic records. 

In all cases, a minimum of three samples were collected intraoperatively (periprosthetic tissues, synovial fluid and sonication fluid) and passed for mycological culturing. The microbiological examination was performed in the laboratory with standard methods for fungal pathogens, with the use of a Sabouraud agar (40 g/L dextrose, 10 g/L peptone, 20 g/L agar; pH = 5.6) growth medium and culture for a minimum of seven days. A positive result was considered when the same fungal pathogen was identified in at least 2 samples (according to ICM 2013 and ICM 2018 definitions) and only such cases were included in the analysis. Inconclusive results including only one positive and a mixed polymicrobial result (three or more fungal and bacterial pathogens) were treated as material contamination. All patients received empiric intravenous antibiotics with or without antifungal agents depending on the department’s recommendations and preoperative microbiological results. Empiric medications were switched to the targeted after the confirmation of the culprit pathogens. Oral antifungal/antibacterial therapy was continued for six weeks. The overall success of the treatment was stated as microbial eradication, with functional joint, no subsequent surgical intervention for infection after revision TJA surgery and no occurrence of PJI-related mortality [30].

### 2.2. Literature Review

**Figure 1:**
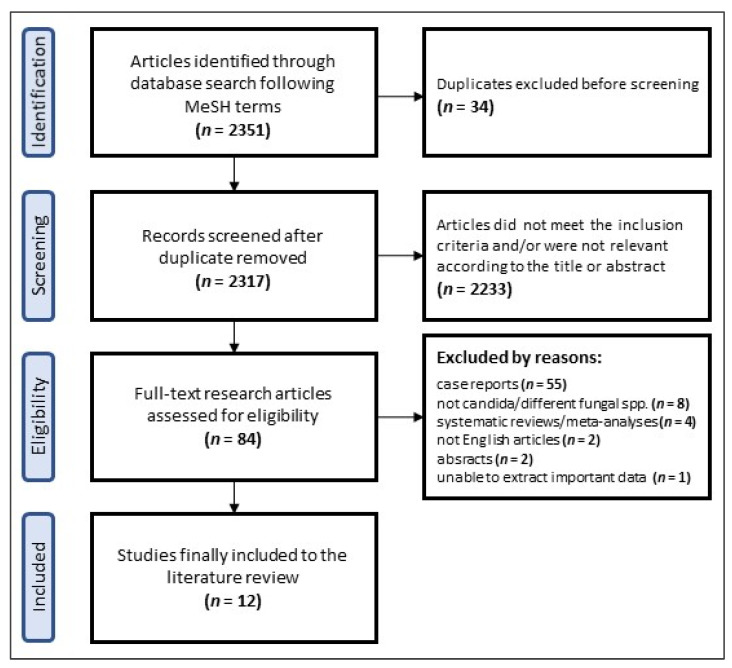
PRISMA (Preferred Reported Item for Systematic review and Meta-Analysis) flow diagram of the articles extracted from the electronic databases following the key MeSH terms.

Research following the MeSH terms: “candida” AND “periprosthetic” OR “prosthetic” AND “joint” AND “infection” was conducted in the PubMed (141 results), Google Scholar (98 results) and Scopus (2112 results) databases on the 10 April 2022. The review was performed according to Preferred Reporting Items for Systematic reviews and Meta-Analyses (PRISMA) guidelines [31]. All titles and abstracts since 1989 were screened for the selection criteria and, subsequently, full-text documents were reviewed. Case reports (reports of three cases and fewer), studies describing infections caused by different fungal species than *Candida*, those involved other joints than hip and knee, no possibility to extract key data of patients with chronic *Candida* PJIs, systematic reviews and meta-analyses were excluded from the analysis. Additionally, only articles in the English language and those with the full-text available were included. The following data in terms of patients’ demographics (age, gender), and clinical data (operated joint, treatment protocols, follow-up and success rate) were analyzed. The corresponding PRISMA flow diagram is presented in Figure 1.

### 2.3. Statistical Analysis

Statistical analysis was done with StatSoft Statistica 13.1 (Tibco Software Inc., Palo Alto, CA, USA). A Shapiro-Wilk test was performed to verify data distribution. For categorical variables, a Fisher’s exact test was used. Continuous variables were analyzed using the Mann-Whitney U test for non-parametric data. *p* values <0.05 were considered as statistically significant.

## 3. Results

### 3.1. Case Series

Eight patients (3 males and 5 females; 2 hips and 6 knees PJIs) met the basic inclusion criteria (Table 1). The median age was 72.5 (Interquartile Range; IQR 61.8–81) and the BMI was 30.7 kg/m^2^ (IQR 29.3–31). The median time from primary TJA to PJI diagnosis was 86.9 months (IQR 24–154). Significant risk factors of fungal PJI were confirmed in five cases (62.5%). The two-stage surgical treatment protocol was applied to seven patients. One patient who underwent one-stage treatment (number 4 in Table 1) was lost after a two-month follow-up. The median follow-up since definitive surgical treatment to the last outpatient visit was 18 months (IQR 7.5–25.5). None of the patients had pain or other clinical signs of infection at the last follow-up visit. All of them presented normal CRP serum concentration and ESR at that time.

The pathogens of PJI in the study group were: *Candida albicans* (3 cases; 37.5%), *Candida parapsilosis* (3 cases; 37.5%), *Candida glabrata* (1 case; 12.5%) and *Candida krusei* (1 case; 12.5%). In one case (number 1 in Table 1), a methicillin-sensitive coagulase-negative *Staphylococcus* (MSCNS) bacterial co-infection was noted. The median CRP serum concentration assessed directly before the surgery for PJI was 8.3 mg/L (IQR 5.5–30.4), and the median ESR was 41 mm/h (IQR 19–58.8). According to the ICM 2018 definition, four patients had normal values of CRP serum concentration (<10 mg/L) and three patients had normal values of ESR (<30 mm/h) [29]. 

**Figure 2:**
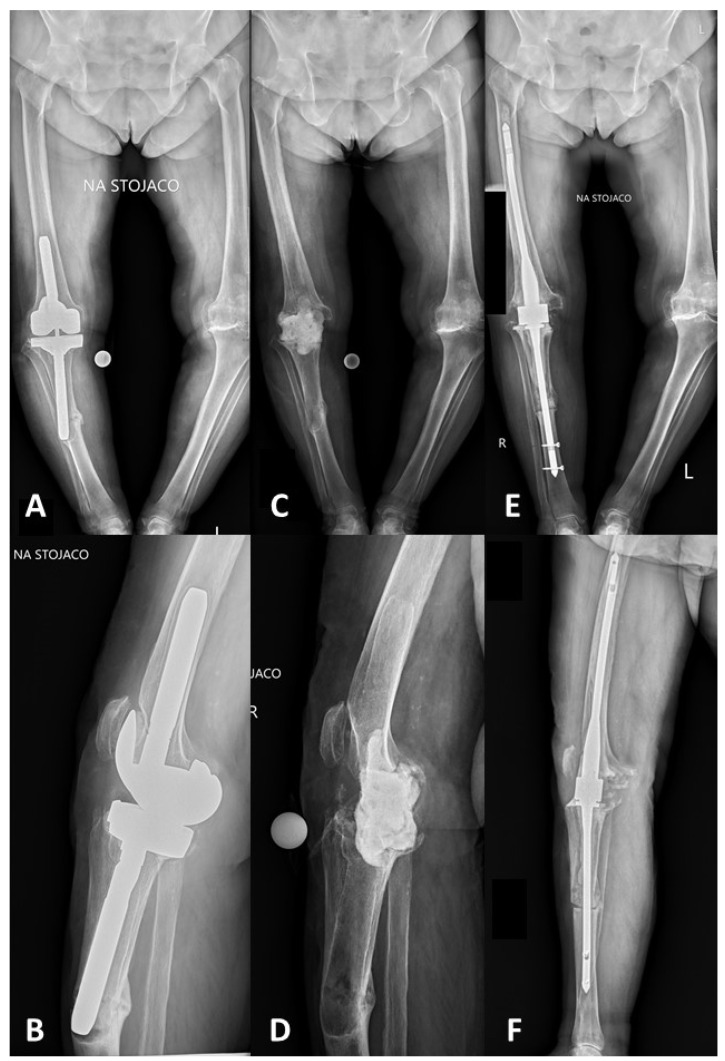
Radiographs of the patient (patient no. 1 in Table 1) with confirmed fungal PJI caused by *Candida parapsilosis*. (**A**) Preoperative AP long-standing radiograph and (**B**) lateral knee radiograph. (**C**) Long-standing AP and (**D**) lateral radiographs after revision surgery and static spacer implantation contained antibiotics. (**E**) Long-standing AP and (**F**) lateral radiographs after knee arthrodesis using a dedicated Charfix2 FN intramedullary nail (ChM, Lewickie, Poland).

The overall success of the treatment was noted in four patients (50%). Three patients underwent knee arthrodesis with the use of an intramedullary nail (Figure 2) and one patient had a resection hip arthroplasty procedure. 

### 3.2. Literature Review

A total of 12 studies comprising 153 cases of *Candida* PJI were included in the final analysis (Table 2) [12,21,22,23,24,25,26,32,33,34,35,36]. The number of hip-to-knee PJI was 86 (54.8%) to 67 (46.2%), respectively. Gender was identifiable in 135 patients, and there were 61 males (45.2%) and 74 females (54.8%). In 10 studies the analysis of risk factors was performed, and among 106 patients in 77 (72.6%) significant comorbidities, fungal PJI were confirmed. One hundred and fifty patients reached a postoperative follow-up (two were lost in follow-up and one refused further treatment), ranging from 13.8 to 72 months.

Regardless of the applied treatment method, the overall success rate was 65.5%. Different surgical protocols were extracted and analyzed in 145 patients from all cohorts. In the one-stage strategy, a 75% (21/28 patients) success rate was noted. The therapeutic success of the two-stage protocol was calculated at 65.3% (47/72). Statistically significant differences in the success rate between these two treatment strategies were not observed (*p* = 0.47). Patients who underwent debridement, antibiotics, and implant retention (DAIR) or three-stage strategies achieved 80% (4/5) and 75% (9/12) success rates, respectively. In patients who underwent resection arthroplasty as the first-line approach, the results differed among the studies depending on the applied definition of therapeutic success by the authors, and in 14/26 patients (53.8%) it was noted when fungal infection was eradicated without joint pain after the surgery. In addition, one patient was qualified for non-surgical treatment and one for arthrodesis as a final treatment. In these two cases, therapeutic success was not achieved. The success rate of the treatment was significantly higher for the knees (77.6%) than for the hips (58%) according to the analysis based on 127 patients. 

**Figure 3:**
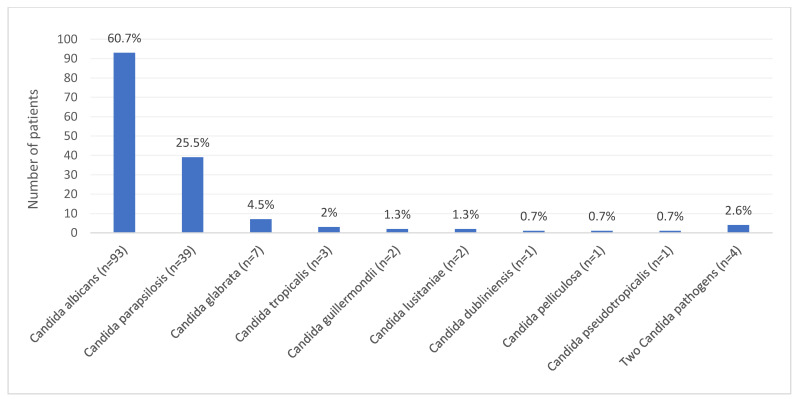
Patients with *Candida* PJIs among the 12 studies included in the review.

The most frequent pathogen was *Candida albicans* (93 cases; 60.7%), followed by *Candida parapsilosis* (39 cases; 25.5%) and *Candida glabrata* (7 cases; 4.5%). In three cases *Candida tropicalis* (2%) was the culprit pathogen. In two cases *Candida guillermondii* (1.3%), or *Candida lusitaniae* (1.3%), and in one case *Candida dubliniensis* (0.7%), *Candida pelliculosa* (0.7%) and *Candida pseudotropicalis* (0.7%) were confirmed. Two *Candida* strains in four cases (2.6%) were identified (Figure 3). Bacterial co-infection was confirmed in 84 patients (54.9%). 

Considering only the most common *Candida* species, in 73 cases of *Candida albicans* and 36 cases of *Candida parapsilosis*, the success rate was 60.1% and 83.3%, respectively, with a statistical significance (*p* = 0.03) (Table 3). The one-stage approach was not statistically more favorable for the treatment of *Candida albicans* or *Candida parapsilosis* (*p* = 0.14). However, for the treatment of *Candida parapsilosis* PJIs, the two-stage protocol was statistically more effective in comparison to the one-stage (*p* = 0.02). Similarly, analyzing the two-stage strategy, better results were achieved for *Candida parapsilosis* than for *Candida albicans* (*p* = 0.04). In the case of joint localization, *Candida albicans* was a significantly more frequent cause of hip PJI and *Candida parapsilosis* of the knee (*p* < 0.01). The risk factors of PJI were more often noted in patients with *Candida albicans* (78%) infections than *Candida parapsilosis* (52%; *p* = 0.03).

## 4. Discussion

Despite the fact that PJIs caused by a *Candida* species are uncommon complications of TJA, they remain a diagnostic and therapeutic challenge for orthopedic surgeons [16,17]. That is why we present an up-to-date review of the literature as well as our single orthopedic center’s cohort of patients with PJI caused by the *Candida* species. Fungal PJI occur more frequently in non-immunocompetent patients with chronic diseases such as rheumatoid arthritis, diabetes mellitus, malignancy, or concomitant bacterial coinfections that cause additional therapeutic problems [6]. These trends were observed in our small cohort as well as in the literature. Additionally, the rarity of occurrence, low-grade development and necessity for performing extended mycological culturing beyond the bacteriological makes diagnostics difficult. In 72.6% of analyzed patients, a minimum of one risk factor of fungal PJI was confirmed. High-risk comorbidities were statistically more often observed in patients infected with *Candida albicans* than *Candida parapsilosis*. Similar frequencies from 50% to nearly 80% have been reported for different fungal PJIs (not only *Candida* species) by the other authors who reviewed and analyzed the literature data [6,8,36]. Moreover, we agree that the risk factors not only predispose to infection but may also influence the treatment outcomes of fungal PJIs and should be minimized before the endoprosthesis reimplantation [6,26].

Clinical outcomes of fungal PJIs also depend on several other important factors such as surgical protocol (e.g., DAIR, one- or two-stage), type of isolate and duration of the antifungal treatment [35]. Currently, a two-stage surgical strategy is the most favorable choice in most institutions, which seems to be a more cautious approach due to a biofilm formation on the implant surface containing fungi with or without bacterial pathogens. The biofilm makes the culprit pathogens highly resistant to antimicrobial agents as well as to the host’s immune system [15]. Contrary to this, analyzing all cases included in the hereby literature review of *Candida* PJIs and according to the definition of therapeutic success applied by specific authors, the results of the one-stage protocol insignificantly exceeded two-stage strategies. Ueng et al. reported a 50% success rate in the treatment of fungal PJI caused by *Candida* [35]. They defined success as a well-functioning joint without the infection relapse after prosthesis replantation during a two-year follow-up. These results are in line with ours, whereby when using the same criteria we achieved a 50% success rate. The best results with a 100% cure rate were obtained by Kim et al., who applied a two-stage protocol to 9 patients after TKA. They have used spacers containing amphotericin B, vancomycin and, optionally, cefazolin and tobramycin [12]. Similarly, Phelan et al., who decided to perform implant removal in their group of four cases and did not make an attempt to replantation [32], Kuiper et al. noted the lowest treatment success (28.6%) [36]. From a group of eight patients, two were fully cured, three underwent resection arthroplasty and two presented decreased outcomes. One patient refused further treatment and was not analyzed.

The overall success rate of the treatment was significantly higher (*p* = 0.03) for *Candida parapsilosis* (83.3%) than for *Candida albicans* (60.3%). Regarding the treatment strategy, the one-stage approach was not favorable for the treatment of *Candida albicans* or *Candida parapsilosis*. However, the two-stage protocol was statistically more effective for *Candida parapsilosis* PJI in comparison to the one-stage protocol (*p* = 0.02) and in comparison to the two-stage treatment results of *Candida albicans* (*p* = 0.4). Similar results were received by Karczewski et al., who compared albicans and non-albicans strains causing PJIs [24]. They reported an 80% of success rate for the non-albicans and 56.3% for albicans PJIs.

The systemic administration of antifungal drugs is a fundamental support for surgical PJI therapy. According to the ICM 2018, fluconazole should be used as the treatment of choice due to susceptible fungi including the most frequent *Candida* species [20]. Several studies recommend amphotericin B, which is likely to be less tolerated [16,20]. Despite the fact that fluconazole and amphotericin B are the most frequently used antifungals, their antibiofilm activity is limited, contrary to the echinocandins and liposomal form of amphotericin B [15]. Therefore, the choice of systemic and local antifungal drugs should be strongly related to the surgical strategy. In our cohort, four patients received fluconazole (50%) postoperatively; however, the decision was based on mycogram, neither ICMs recommendations. Patients continued with the oral administration of antifungal agents for a minimum of six weeks before making the decision of reimplantation, which is currently recommended by the ICM 2018 as well. Additionally, in two cases antibiotics were ordered together with antifungal agents postoperatively. In one case this was, due to a positive result of bacterial culturing in sonication (MSCNS), and in one case it was due to a positive result of previous culturing despite the negative intraoperative. Mixed fungal/bacterial co-infections were not found to be more frequent in our small cohort, contrary to the literature, which found one or more bacterial pathogens in 54.9% of cases. For this reason, the administration of the additional antibiotics should be considered in fungal PJIs, and treatment should be initiated upon confirmation of bacterial coinfection.

Other studies which did not meet the inclusion criteria for our analysis were also in line with the results of our systematic review results. Gao et al. observed 55.6% of mixed infections (10 out of 18 cases of fungal PJIs) [18]. A higher percentage of mixed infections in the failure group (80%) was noted than in the success group (46.2%); however, did not differ significantly (*p* = 0.314). We agree with these authors’ conclusion that additional antibiotics should not be routinely administered when the absence of bacterial infection is confirmed. Similarly, Kuo et al., who analyzed the two-stage treatment protocol, observed 51.7% of bacterial co-infections [19]. They compared the results of all fungal infections to bacterial PJIs and fungal infections alone to mixed fungal/bacterial PJIs. Treatment failure was significantly higher in fungal (58.6%) than in non-fungal PJIs (28%; *p* < 0.001), as well as the revision index (51.7% vs 29.6%; *p* = 0.01). In the case of fungal PJIs (alone and mixed), there were no statistically significant differences in the failure rate between the groups (42.9% vs 73.3%; *p* = 0.139), but they were statistically different in terms of the revision index (28.6% vs 73.3%; *p* = 0.027). 

The largest review analyzing various fungal PJIs included 32 articles and was performed on data collected from 286 cases [7]. However, this study included PJIs after THA (139 cases), TKA (142 cases), total elbow (two cases) and shoulder (three cases) arthroplasties. Moreover, different types of articles and fungal strains (not only *Candida*) were included without the possibility to extract data concerning the *Candida* species. The important finding that stayed in line with our results was the overall success rate, which did not differ significantly depending on the surgical strategy. When the two-stage protocol was used, a 65% success rate was revealed, and this was 59% after the one-stage treatment (*p* = 0.485). Bacterial co-infection was confirmed in 30.4% of cases and, unfortunately, the connection between this factor and the success rate, as well as in the case of specific fungal strains was not analyzed.

The evident limitations of our study are the retrospective character and the small group of patients included in the analysis. These are the main reasons that make the analysis vulnerable to statistical bias. Due to the rarity of *Candida* PJIs, a single-center, large-cohort study with prospectively collected data seems to be challenging. The paucity of clinical and demographic data in some of the reviewed papers might have influenced our statistical analysis. However, to our knowledge, this is the largest review and analysis summarizing the clinical outcomes of treatment of the *Candida* PJIs based on the case series studies. 

## 5. Conclusions

In conclusion, *Candida* PJIs were rare complications of THA and TKA in a single orthopedic center, with their success rate of treatment at 50%. According to the literature review, one- or two-stage surgical strategies for the treatment of PJIs caused by all *Candida* species presented with similar results. The two-stage surgical protocol revealed to be more effective in the treatment of PJIs due to *Candida parapsilosis*. The treatment of *Candida* PJI following TKA presented with better outcomes than those following THA. Similarly, the two-stage treatment applied for *Candida parapsilosis* indicates a higher therapeutic success rate than for *Candida albicans* strains. However, these findings need prospective, multicenter verification and to receive the preoperative results of mycological culturing.

## Figures and Tables

**Table 1:** Patients’ characteristics included in the analysis.

No.	Age (Years)	Sex	Joint	BMI (kg/m^2^)	Risk Factors	Time from TJA to PJI Diagnosis (Months)	Preoperative CRP (mg/L)	Preoperative ESR (mm/h)	Pathogen	Material for Mycological Culturing	Surgical Protocol	Intravenous Antibacterial/Antifungal Agents	Oral Antibacterial/Antifungal Agents	Follow-Up (after Definitive Procedure) [Months]	Final Outcome
1	83	F	Knee	37.39	None	74	5.4	10	*Candida parapsilosis*, MSCNS	Tissues (+) Synovial fluid (+) Sonication (+)	Two-stage	CFX + AMC	FLU + CFX	18	Nail arthrodesis
2	68	F	Knee	30.47	Sepsis due to renal failure before the PJI	28	1.9	22	*Candida glabrata*	Tissues (+) Synovial fluid (+) Sonication (+)	Two-stage	VAN + AMB + MYC	ITR	10	Prosthesis replantation
3	79	F	Knee	30.48	Psoriathic arthritis	150	64.6	103	*Candida albicans*	Tissues (+) Synovial fluid (−) Sonication (+)	Two-stage	AMC + CEF + FLU	FLU + AMO/CLA	37	Nail arthrodesis
4	63	M	Hip	30.99	Alcohilizm	158	6.2	10	*Candida albicans*	Tissues (+) Synovial fluid (−) Sonication (−)	One-stage	CFX + AMC	FLU	2 (lost follow-up)	Prosthesis replantation
5	77	F	Knee	25.65	None	9	19	44	*Candida albicans*	Tissues (+) Synovial fluid (−) Sonication (+)	Two-stage	CFX + AMC	FLU	22	Prosthesis replantation
6	84	M	Hip	31.07	None	10	86.3	135	*Candida krusei*	Tissues (+) Synovial fluid (−) Sonication (+)	Two-stage	CFX + AMC	ITR	10	Resection arthroplasty
7	51	F	Knee	24.01	Rheumatoid arthritis	26	5.5	42	*Candida parapsilosis*	Tissues (+) Synovial fluid (−) Sonication (n/d)	Two-stage	CFX + AMC	KET	36	Nail arthrodesis
8	58	M	Knee	35.44	After yersinia and chlamydia infections	12	10.4	40	*Candida parapsilosis*	Tissues (+) Synovial fluid (−) Sonication (+)	Two-stage	AMB + MYC	ITR	6	Prosthesis replantation

FLU—Fluconazole; ITR—Itraconazole; KET—Ketoconazole; VAN—Vancomycin; CEF—Cefuroxime; AMB—Amphotericin B;. MYC—Micafungin; AMC—Amikacin; CFX—Ceftriaxone; AMO/CLA—Amoxycillin/Clavulanic acid; MSCNS—methicillin-sensitive coagulase-negative *Staphylococcus*. The column entitled “Material for mycological culturing”: (+ and −) concerns the positive and negative results of culturing. In all cases, from two to four tissue samples were collected for microbiological examination.

**Table 2:** General overview of the analyzed studies.

	Country	Number of Patients (Male/Female)	Mean Age (Years)	Joint	Mean Preoperative CRP (mg/L)	Bacterial Co-Infection	Risk Factors	Mean Follow-Up (Months)	Therapeutic Success/ Treatment Protocol (n/m) %	Overall Success Rate **
Darouiche et al. (1989) [32]	USA	4 (3/1)	63.5	K—1 (25%) H—3 (75%)	N/A	Yes—0 No—4 (100%)	Yes—2 (50%) No—2 (50%)	13.8	Resection arthroplasty—(0/4) 0%	0%
Phelan et al. (2002) [33]	USA	4 (2/2)	72.3	K—1 (25%) H—3 (75%)	N/A	Yes—0 No—4 (100%)	Yes—4 (100%) No—0	52.8	Two-stage—(4/4) 100%	100%
Dutronc et al. (2010) [34]	France	7 (3/4)	72	K—4 (57.1%) H—3 (42.9%)	98.1	Yes—0 No—7 (100%)	Yes—5 (71.4%) No—2 (28.6%)	30	No surgery—(0/1) 0% DIAR (1/1)—100% Two-stage—(1/3) 33.3% Arthrodesis—(0/1) 0% Resection arthroplasty—(1/1) 100%	42.9%
Ueng et al. (2013) [35]	Taiwan	16 (12/4)	55.4	K—9 (52.9%) H—7 (47.1%)	N/A	Yes—8 (50%) No—8 (50%)	Yes—11 (68.8%) No—5 (31.2%)	41	Two-stage—(8/9) 88.9% Resection arthroplasty—(3/7) 42.9%	68.8%
Kuiper et al. (2013) [36]	Netherland	8 (2/6)	72.8	H—8 (100%)	47	Yes—0 No—8 (100%)	Yes—7 (87.5%) No—1 (12.5%)	30.4	Two-stage—(2/7) 28.6% ***	28.6% ***
Klatte et al. (2014) [22]	Germany	10 (6/4)	67.8	K—4 (40%) H—6 (60%)	22	Yes—6 (60%) No—4 (40%)	Yes—7 (70%) No—3 (30%)	72	One-stage—(9/10) 90%	90%
Ji et al. (2017) [21]	China	11 (4/7)	66.5	K—7 (63.6%) H—4 (36.4%)	N/A	Yes—8 (72.7%) No—3 (27.3%)	Yes—6 (54.5%) No—5 (45.5%)	60	One-stage—(9/11) 81.8%	81.8%
Kim et al. (2018) [12]	South Korea	9 (1/8)	76	K—9 (100%)	22.6	Yes—7 (77.8%) No—2 (22.2%)	Yes—4 (44.4%) No—5 (55.6%)	66	Two-stage—(9/9) 100%	100%
Theil et al. (2019) [26]	Germany	26 (10/16)	71.9	K—8 (30.8%) H—18 (69.2%)	N/A	Yes—13 (50%) No—13 (50%)	Yes—24 (92.3%) No—2 (7.7%)	33 *	One-stage—(0/2) 0% Two-stage—(10/24) 41.7%	38.5%
Saconi et al. (2020) [25]	Brazil	11 (5/6)	65.1	K—5 (45.5%) H—6 (54.5%)	312	Yes—6 (54.5%) No—5 (45.5%)	Yes—7 (63.6%) No—4 (36.4%)	41.7	DAIR—(1/1) 100% *** One-stage—(2/3) 66.7% *** Two-stage—(1/1) 100% Resection arthroplasty—(4/4) 100%	88.9% ***
Enz et al. (2021) [23]	Germany	18 (N/A)	70.2	K—4 (22.2%) H—14 (77.8%)	N/A	Yes—14 (22.2%) No—4 (77.8%)	N/A	N/A	DAIR—(1/1) 100% **** Two-stage—(5/7) 71.4% **** Resection arthroplasty—(3/5) 60% ****	72.2% ****
Karczewski et al. (2022) [24]	Germany	29 (13/16)	71	K—15 (51.7%) H—14 (48.3%)	51.7	Yes—22 (68.2%) No—7 (31.8%)	N/A	33	DIAR— (1/2) 50% One-stage—(1/2) 50% Two-stage—(7/8) 87.5% Three-stage—(9/12) 75% Resection arthroplasty—(3/5) 60%	72.4%

K—knee; H—hip; N/A—non-available to extract data; DAIR—debridement, antibiotics and implant retention; * Median value; ** Success was recognized in accordance with the definition used by the authors; *** Excluded patients that refused further treatment or were lost in follow-up; **** results were presented partially (for 13 patients).

**Table 3:** Comparison of *Candida albicans* and *Candida parapsilosis* fungal PJIs. Data were extracted from the literature included in the systematic review. Continuous values are presented as median with Interquartile Range (IQR).

	*Candida* *albicans*	*Candida* *parapsilosis*	*p*-Value
Males/Females **(#)**	33/24	17/18	0.4 *
Hip/Knee	50/23	9/27	<0.01 *
Median age (years)	73 (62–79)	71 (65–77)	0.88 **
Bacterial co-infection (%)	43 (58.9%)	17 (47.2%)	0.3 *
Risk factors (no. of patients; %) **(##)**	32 (78%)	13 (52%)	0.03 *
Type of risk factors **(##)** - Diabetes mellitus - Prior *Candida* infection - Systemic lupus erythromatosus - Immunosuppression - Malignancy - Myelodysplastic syndrome - Prior PJI - Chronic obstructive pulmonary disease - More than one risk factor - None	7 2 2 1 1 1 - - 18 9	6 - - - 1 1 1 1 3 12	
Treatment protocol [no. of patients] **(#)** - DAIR - One-stage - Two-stage - Three-stage - Resection arthroplasty/arthrodesis	2 14 20 5 16	0 8 18 3 6	
Overall success rate (*n*; %)	44/72 (60.3%) —one patient lost to follow-up	30/36 (83.3%)	0.03 *
Success rate depending on treatment protocol [no. of patients, (%)] **(#)** - DAIR - One-stage - Two-stage - Three-stage - Resection arthroplasty/arthrodesis	1 (50%) 12 (85.7%) 13 (65%) 4 (80%) 2 (66.7%)—one patient lost to follow-up	- 4 (50%) 17 (94.4%) 2 (66.7%) 6 (100%)	- 0.14 * 0.04 * - -

* Fisher’s exact test; ** Mann-Whitney U test. **(#)** Unable to extract data from 16 cases of *Candida albicans* and one case of *Candida parapsilosis* PJIs. **(##)** Unable to extract data from 32 cases of *Candida albicans* and 11 cases of *Candida parapsilosis* PJIs.

## Data Availability

Not applicable.
